# Veterinary fluoroquinolones as emerging contaminants in marine environments: *In vitro* study of biochemical responses in subcellular fractions of the Mediterranean mussel (*Mytilus galloprovincialis*)

**DOI:** 10.1016/j.heliyon.2024.e40467

**Published:** 2024-11-17

**Authors:** J. Giannessi, L. De Marchi, V. Meucci, L. Intorre, G. Monni, M. Baratti, C. Pretti

**Affiliations:** a Department of Veterinary Sciences, Università di Pisa, Via Livornese (lato monte), Pisa, San Piero a Grado, 56122, Italy; b Research Institute on Terrestrial Ecosystems, IRET-CNR, Via Madonna del Piano 10, Firenze, Sesto Fiorentino, 50019, Italy; c Interuniversity Consortium of Marine Biology and Applied Ecology "G. Bacci” (CIBM), Viale N. Sauro 4, Livorno, 57128, Italy

**Keywords:** Fluoroquinolones antibiotics, *Mytilus galloprovincialis*, Subcellular responses, S9-fractions, Biomarkers

## Abstract

Fluoroquinolone antibiotics (FQs) are emerging pollutants frequently detected in aquatic environments. However, their impact on marine invertebrates remains underexplored. This study investigated the responses at subcellular level in the Mediterranean mussel (*Mytilus galloprovincialis*) exposed to three veterinary FQs, marbofloxacin (MARB), sarafloxacin (SARA), and difloxacin (DI), at concentrations considered relevant to environmental conditions. The assessment focused on the digestive gland and gills, employing *in vitro* assays to evaluate antioxidant defenses, biotransformation, and neurotransmission enzyme activities, as well as their effects on membrane lipids, proteins, and DNA integrity. Results revealed a general decline in antioxidant defenses and compromised DNA integrity in both tissues. Additionally, exposure to MARB and DI led to an alteration in detoxification capacity in the gills, along with an increased content of carbonylated proteins. Conversely, the digestive gland exhibited a significant inhibition of acetylcholinesterase activity. These findings suggest potential neurotoxic and genotoxic impacts of these antibiotics on non-target species, as well as an associated oxidative effect.

## 1 Introduction

Fluoroquinolones (FQs) antibiotics are classified as broad-spectrum antimicrobial agents, recognized for their bactericidal efficacy against a variety of pathogenic bacteria. As a result, in the last years, they have seen increased utilization in human medicine and veterinary applications such as livestock and aquaculture. FQs act by inhibiting the activity of essential bacterial enzymes, namely type-II (DNA gyrase) and type-IV topoisomerases. These enzymes are crucial for the replication and transcription processes in prokaryotes, leading to the potent bactericidal activity of FQs [[1], [2], [3]].

The efficacy of FQs in treating infections affecting multiple systems, including the urinary, gastrointestinal, abdominal, and respiratory systems in both animals and humans, underscores their importance [4,5]. Classified as critically important antibiotics for humans by the World Health Organization (WHO) and for animals by the World Organisation for Animal Health (WOAH), FQs play a crucial role in combating bacterial infections in both sectors.

The introduction of first-generation fluoroquinolones, exemplified by nalidixic acid, and second-generation FQ antibiotics (e.g. ciprofloxacin, enrofloxacin, ofloxacin, and norfloxacin) has left a significant imprint on the pharmaceutical market [6]. This progression has extended to the development of third and fourth-generation FQs, characterized by the incorporation of various substituents at different positions on the pharmacophore. This modification aims to enhance the broad-spectrum activity of these antibiotics, resulting in an ever-increasing efficacy [3].

The increased production and usage of FQ antibiotics have led to their increased presence in the environment, primarily through the discharge of byproducts and effluents from various sources such as municipal, agricultural, and industrial wastewater.

This is due to a significant portion (ranging from 30 % to as much as 90 %) of antibiotics administered to humans and animals being discharged into wastewater through urine and feces, often in an unaltered state [7,8]. Conventional wastewater treatment methods display only partial efficacy in removing or breaking down these substances [9,10], resulting in their release into freshwater environments and until reaching the sea, posing a threat to aquatic ecosystems.

Recently, FQs have been identified as a major group of pollutants in aquatic environments, with concentrations detected between ng/L and μg/L [1]. Once introduced into these ecosystems, FQs can adversely affect non-target organisms, disrupting crucial bacterial mechanisms and processes. This disruption fosters the development of resistance in microbial communities, thereby contributing to the rise of antibiotic resistance, which represents a severe public health concern [11,12]. In addition, several studies highlight the detection of antibiotics (e.g., tetracyclines, sulfonamides), including FQs, in marine bivalves, indicating a significant risk of bioaccumulation in these organisms [13]. FQs are notably recognized for their ecotoxicological effects. Key environmental toxicology mechanisms associated with FQs include the production of free radicals, DNA fragmentation, mitochondrial damage, apoptosis, and oxidative stress. These impacts have been observed across a wide range of concentrations in various organisms, including bacteria, algae, invertebrates, and plants [1,14,15].

However, there remains a gap in understanding the subcellular biomarker responses of non-target organisms, particularly marine bivalves, to FQ antibiotics.

To address this issue, this study focused on examining the differential responses of *M. galloprovincialis’* target tissues (gills and digestive gland) to FQs exposure. Specifically, we conducted an *in vitro* study using environmentally relevant concentrations of MARB, SARA, and DI.

Antibiotics, like most drugs, can intentionally exert biological effects, distinguishing them from other chemicals where toxicity typically manifests as an unintended side effect of their primary function [16]. These FQs, categorized within the second generation, are specifically used for veterinary applications [[17], [18], [19], [20]]. They demonstrate bactericidal activity against a wide spectrum of aerobic gram-negative and certain gram-positive bacteria affecting cattle, swine, poultry and aquacultured species (WHO, 1998). As a result, they represent a significant source of environmental contamination. Unlike other pharmaceuticals, antibiotics such as FQs have notable gaps in research concerning their toxicological effects on non-target organisms [21]. Veterinary-specific FQs, in particular, received less attention compared to other molecules like ciprofloxacin and enrofloxacin, but they remain crucial due to their continued detection in aquatic environments. This is largely due to livestock farming, runoff from agricultural areas, and discharge from aquaculture activities [i.e. [16]. Focusing on these compounds aligns with the broader need to investigate emerging contaminants and to better understand the potential risks posed by this entire class of antibiotics.

Given that exposure to FQs may disrupt multiple biochemical pathways, this research aimed to assess the toxicity effects of MARB, SARA, and DI on target tissues in marine bivalves. Based on prior assumptions, we used an *in vitro* approach to investigate how FQs interact with antioxidant enzymatic defenses, metabolic processes, and the stability of membrane lipids and proteins. We also evaluated their effects on neurotransmission and DNA integrity.

## 2 Materials and methods

### 2.1 Chemicals preparation

Marbofloxacin (CAS:115550-35-1), sarafloxacin (CAS:91296-87-6), and difloxacin (CAS: 91296-86-5) with a purity ≥98 % were purchased from Sigma-Aldrich (Milan, Italy). Three concentrations were chosen for testing each contaminant (5, 50, and 500 ng/L), which previous studies have identified as environmentally relevant in the marine ecosystem [9,22,23].

Stock solutions at a concentration of 0.1 g/L were initially prepared in ultra-pure deionized water, followed by serial dilution to obtain the required final concentrations within the reaction mixture.

### 2.2 Animals collection and acclimation

Adult mussels of the *Mytilus galloprovincialis* species were gathered from a mussel farming facility located along the northwestern coast of the Tyrrhenian Sea in La Spezia, Italy. Following the collection, the mussels were promptly transferred to the laboratory while maintaining wet conditions. Organisms were given a 10-day acclimation period in 20 L aquariums (N = 20 mussels *per* aquaria) containing naturally filtered seawater (NFSW) with a pore size of 0.45 μm. During acclimation, conditions were kept stable at a temperature of 20.0 ± 1.0 °C, salinity of 38 ± 1 PSU, pH level at 8.1 ± 0.1, continuous aeration, and a 16:8 h (light:darkness) photoperiod. Throughout this duration, the NFSW was renewed every two days, all the conditions were checked daily and nitrogen levels, including ammonia (NH₃/NH₄⁺), nitrites (NO₂⁻), and nitrates (NO₃⁻), were closely monitored to ensure optimal water quality (respectively, ≤0,05, ≤0.02, ≤0.20 ppm). Additionally, mussels received feeding three times a week with AlgaMac Protein Plus (Aquafauna Bio-Marine, Inc.), at an approximate rate of 150,000 cells *per* animal *per* day.

### 2.3 Sample processing and preparation of subcellular fractions-S9

Following the acclimation phase, 40 mussels with comparable sizes (shell lengths ranging from 6.0 to 7.0 cm) and same sex (males) were chosen. Each individual was opened and dissected with sterile surgical instruments to separately collect digestive gland (DG) and gill (G) tissues. The freshly excised tissues were combined into pools, and their collective weight was measured for the subsequent preparation of subcellular S9 fractions as follows. Each pool was homogenized with marker-specific buffers using an Elvehjem Potter. Specifically, for all assays, a phosphate buffer pH 8.0 (composed of K_2_HPO_4_, 50 mM KH_2_PO_4_, 50 mM K_3_PO_4_) at a ratio of 1:4 (v/v) was utilized, while for the DNAssb assessment a Tris-EDTA (TE) buffer pH 7.4 (10 mM Tris-HCl, 1 M EDTA) in a ratio of 1:10 (v/v) was employed. The homogenates were centrifuged at 9000 g for 20 min at 4 °C, except for those in TE buffer, which were not centrifuged to preserve the integrity of DNA in the sample. The obtained supernatants (S9 fractions) were aliquoted and quickly frozen in liquid nitrogen before being stored at −80 °C until needed. This approach preserves biomaterial and reduces reliance on live organisms. Studies have shown that enzyme integrity and activity are largely maintained during freezing and thawing processes if handled properly. Most enzymes, despite the loss of cell viability, preserved their activity and remained functional for *in vitro* toxicity assessments [24].

Before experimental exposure, the total content of proteins for each pool of tissue (DG and G) was measured using the Lowry et al. method [25].

### 2.4 *In vitro* exposure

*In vitro* experiments were performed exposing S9 fractions of DG and G to selected antibiotics, following the guidelines outlined by the OECD (2018), with slight modifications. The digestive gland and gills were selected as key target tissues in ecotoxicological studies due to their relevant roles in assessing biochemical effects. Gills are the primary site of interaction with environmental contaminants, while the digestive gland plays a central role in the biotransformation of xenobiotics, making both tissues pivotal in detoxification processes and oxidative stress responses [26].

A preliminary experiment was carried out to determine the optimal exposure conditions for the assay, including factors such as protein quantity, incubation time, and temperature (see Fig. S1 in Supplementary materials for details). Based on the findings from this preliminary experiment, the standard protein concentration for *in vitro* exposure to chemicals was established at 1 mg (except for the CbE enzymatic assay, where 0.5 mg/protein was used) and the incubation period was set at 30 min at 25 °C. Following exposure, biochemical parameters were analyzed using 96-well microplates and the measurements were red using a microplate reader, specifically the Synergy HT model from BioTek® Inc.

For each assay, three replicates were used for every antibiotic and at each tested concentration. Additionally, three replicates of each tissue without exposure to the antibiotics served as the negative control group.

### 2.5 Biochemical analysis

The study assessed tissue-specific responses to selected antibiotics through the evaluation of a biomarker battery, including: antioxidant capacity via glutathione peroxidase, (GPx) and superoxide dismutase (SOD) activities; detoxification capacity involving glutathione S-transferase (GST) and carboxyl-esterase (CbE) activities; cellular damage measured by protein carbonylation (PC) and lipid peroxidation (LPO) levels; neurotoxicity evaluated by acetylcholinesterase (AChE) activity; and DNA integrity examined through DNA single-strand break (DNAssb).

#### 2.5.1 Antioxidant defenses

GPX activity was measured using t-butyl hydroperoxide (TBH) as a substrate, following the method by Badary et al. [27], with results expressed in nmol of NADPH oxidized *per* minute *per* mg of protein.

SOD enzyme activity was assessed according to the procedure of Magnani et al. [28], which utilized Cu-Zn SOD to inhibit pyrogallol autoxidation. In this method, the presence of ethylenediaminetetraacetic acid (EDTA) at pH 8.2 leads to a 50 % reduction in autoxidation. Absorbance was recorded at 420 nm initially and again 1 min after adding pyrogallol. Results were expressed in U/mL, where one unit (U) indicates the enzyme amount needed to achieve 50 % inhibition of pyrogallol autoxidation.

#### 2.5.2 Biotransformation capacity

GST enzymatic activity was measured following the method of Habig et al. [29] by tracking the increase in absorbance at 340 nm. This involved monitoring the increase in absorbance at 340 nm, which reflects the conjugation reaction between reduced glutathione (GSH) and CDNB (1-chloro-2,4-dinitrobenzene). Results were reported as nmol *per* minute *per* mg of protein.

CbE activity was determined according to Hosokawa & Satoh [30]. The substrate for these esterases, p-nitrophenyl butyrate (p-NPB), undergoes hydrolysis, producing the chromophore 4-nitrophenol. The formation of 4-nitrophenol was measured over a period of 3 min at 415 nm (with ε = 18mM^−1^cm^−1^). The outcomes was expressed as nmol *per* minute *per* mg of protein.

#### 2.5.3 Cellular damage

LPO levels were measured by quantifying thiobarbituric acid reactive substances (TBARS) through the method outlined by Ohkawa et al. [31]. This method measures malondialdehyde (MDA), a byproduct of polyunsaturated fat breakdown, which reacts with 2-thiobarbituric acid (TBA) to form TBARS. MDA concentration was recorded at 532 nm using an extinction coefficient of 1.56 x 10^5 M⁻^1^cm⁻^1^, with results expressed as nmol MDA *per* gram of fresh tissue (FW).

PC levels were measured following Mesquita et al. [32], involving a reaction between 2,4-Dinitrophenylhydrazine (DNPH) and carbonyl groups. Absorbance was recorded at 450 nm and 750 nm, and results reported in nmol *per* mg of protein.

#### 2.5.4 Neurotoxicity

The enzymatic activity of AChE was determined using Ellman's method [33]. his enzyme hydrolyzes acetylthiocholine iodide (ATChI) as a substrate, which leads to the formation of thiocholine iodide. This product then reacts with 2,2′-dinitro-5,5′-dithiodibenzoic acid (DTNB) to produce 5-thio-2-nitrobenzoate (TNB). The reaction was monitored for 5 min at a wavelength of 412 nm, with the molar extinction coefficient for TNB set at 136,000 mM⁻^1^cm⁻^1^. Results were expressed in terms of nanomoles *per* minute *per* milligram of protein.

#### 2.5.5 DNA integrity

DNA single-strand breaks (DNAssb) were assessed using the Fast Micromethod® [34], which measures DNA integrity under highly alkaline conditions. In this setup, the fluorescent dye Pico488 selectively binds to double-stranded DNA (dsDNA) and proteins, while showing minimal binding affinity to single-stranded DNA (ssDNA). Some modifications were made to the original protocol. The decline in fluorescence intensity of the Pico488-dsDNA complex was monitored over a 10-min interval, with excitation and emission wavelengths set at 485 nm and 520 nm, respectively. Results were expressed as the strand scission factor (SSF), quantifying DNA single-strand breaks as calculated in Equation (1):

(1) $$SFF={-\log}_{10}\frac{Fluorescence\;units\;of\;sample}{Fluorescence\;units\;of\;control}$$

Thus, when SSF = 0, it indicates no additional DNA strand breaks or alkali-labile sites. In contrast, SSF <0 signifies an increasing frequency of strand breaks and alkali-labile sites, reflecting a loss of DNA integrity in the samples. For consistency, the control condition (CTRL) was set to SSF = 0, and values in graphical representations were multiplied by (−1) for clarity.

### 2.6 Data analysis

#### 2.6.1 Statistical analysis

The null hypothesis tested was that MARB, SARA, and DI would have no significant effect on the subcellular fractions of the digestive gland and gills in mussels across all tested concentrations, showing no notable differences between the control and exposed tissues. To test this hypothesis, all experimental data was analyzed using a one-way ANOVA followed by Tukey's post-hoc test to identify any statistically significant variations among the different experimental conditions. Before the analysis, the data was checked for normality using the Shapiro-Wilk test and for homogeneity of variance using the Bartlett test. GraphPad Prism 9.0 Software was used for all statistical analysis, and the results are presented as mean values with standard deviation. Statistically significant differences compared to the control groups are indicated by asterisks: p < 0.05 = ∗, p < 0.01 = ∗∗, and p < 0.001 = ∗∗∗.

#### 2.6.2 Multivariate analysis

The collected data were analyzed using Principal Coordinates Ordination (PCO) with PRIMER v6 [35]. An Euclidean distance similarity matrix was created including all biochemical parameters (SOD, GPX, GST, CE, LPO, PC, AChE, DNAssb) of both exposed tissues to different concentrations of MARB, SARA, and DI. Distances between centroids were then calculated.

Vectors were superimposed on the PCO graph to visually represent the relationships between biochemical responses in the DG and G and the varying concentrations of MARB, SARA, and DI treatments, with only those showing a correlation above 75 % (r > 0.75) included. This analytical method allowed for determining which treatment had the strongest influence on each biochemical parameter and made it possible to cluster treatments based on their proximity in similarity.

## 3 Results

### 3.1 Antioxidant defenses

**Fig. 1:**
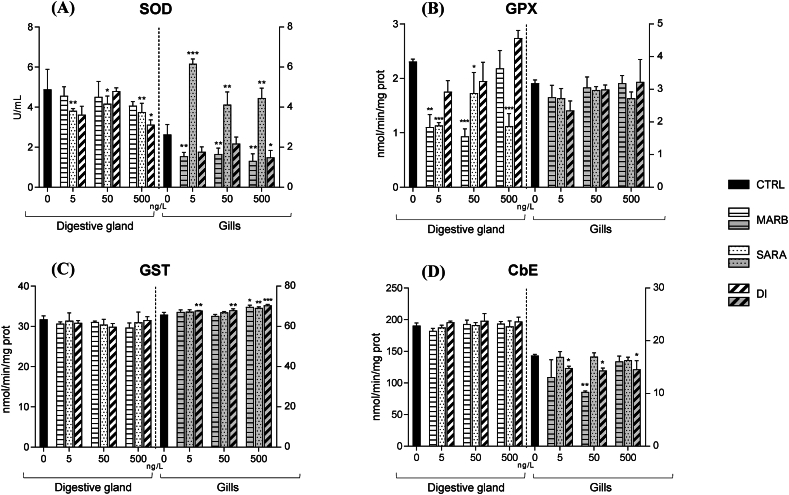
S9-fractions from the digestive gland (DG) and gills (G) of *M. galloprovincialis* were subjected to *in vitro* exposure to concentrations of 0 (CTRL), 5, 50, and 500 ng/L of MARB, SARA, and DI. Mean values (±DS) are provided for different biochemical parameters: superoxide dismutase (SOD, panel A); glutathione peroxidase (GPx, panel B); glutathione-S-transferase (GST, panel C); carboxylesterase (CbE, panel D) activities. Statistical significance from the control is denoted as ∗ for p < 0.05, ∗∗ for p < 0.01, and ∗∗∗ for p < 0.001, as determined by one-way ANOVA followed by Dunnett's test.

Superoxide dismutase (SOD) activity exhibited different patterns in the two analyzed tissues, as depicted in Fig. 1A. In the DG, a significant decrease was observed when exposed to all concentrations of SARA and to the highest concentration (500 ng/L) of DI, whereas MARB had no effect. Conversely, in the G, all tested concentrations of MARB such as 500 ng/L of DI lead to a significant decrease in the enzyme activity. At the same time, SARA showed a significant SOD activity increase when compared to the control condition.

Glutathione peroxidase (GPx) activity was notably inhibited in the DG tissue. Specifically, this inhibition occurred when exposed to the low and intermediate concentrations of MARB (5, 50 ng/L) and all concentrations of SARA. In contrast, no effects on GPx activity were observed in the G tissue when exposed to all tested FQs (Fig. 1B).

### 3.2 Biotransformation capacity

In the DG, there were no significant differences in GST activity between the control group and any of the experimental conditions. In contrast, the gills exhibited a marked increase in GST activity at the highest tested concentrations of MARB and SARA (500 ng/L), as well as at all tested concentrations of DI (Fig. 1C).

The carboxylesterase (CbE) activity did not change in the DG exposed to FQs compared to the unexposed tissue. However, differences in its activity were observed in the G; specifically, there was a decrease in its activity when exposed to the intermediate concentration of SARA (50 ng/L) and to all tested concentrations of DI (Fig. 1D).

### 3.3 Cellular damage

**Fig. 2:**
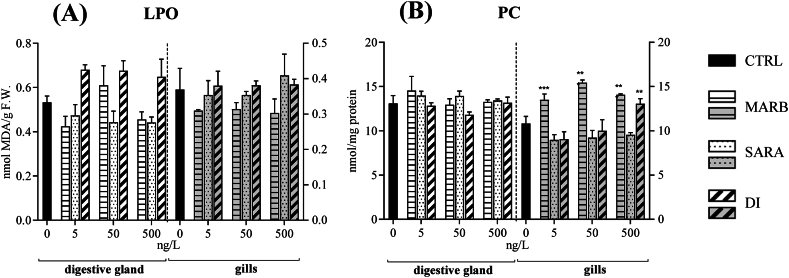
S9-fractions from the digestive gland (DG) and gills (G) of *M. galloprovincialis* were subjected to *in vitro* exposure to concentrations of 0 (CTRL), 5, 50, and 500 ng/L of MARB, SARA, and DI. Mean values (±DS) are provided for lipid peroxidation (LPO, panel A) and protein carbonylation (PC, panel B) levels. Statistical significance from the control is denoted as ∗ for p < 0.05, ∗∗ for p < 0.01, and ∗∗∗ for p < 0.001, as determined by one-way ANOVA followed by Dunnett's test.

As shown in Fig. 2A, there were no significant differences in lipid peroxidation (LPO) across all treatments in either tissue.

No differences were observed in protein carbonylation (PC) levels among treatments in mussels’ DG. Conversely, higher levels of PC were observed in the G tissue exposed to MARB and to the highest concentration of DI (500 ng/L), whereas SARA exhibited no significant effects (Fig. 2B).

### 3.4 Neurotoxicity

**Fig. 3:**
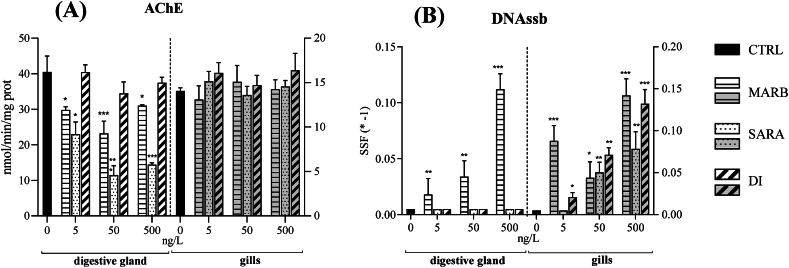
S9-fractions from the digestive gland (DG) and gills (G) of *M. galloprovincialis* were subjected to *in vitro* exposure to concentrations of 0 (CTRL), 5, 50, and 500 ng/L of MARB, SARA, and DI. Mean values (±DS) are provided for acetylcholinesterase (AChE, panel A) activity and DNA single-strand break (DNAssb, panel B). Statistical significance from the control is denoted as ∗ for p < 0.05, ∗∗ for p < 0.01, and ∗∗∗ for p < 0.001, as determined by one-way ANOVA followed by Dunnett's test.

A significant reduction in acetylcholinesterase (AChE) activity was observed in the DG of mussels exposed to MARB and SARA, across all tested conditions. Conversely, in the G tissue, no differences between treatments and control condition were detected (Fig. 3A).

### 3.5 DNA integrity

A statistically significant increase in DNA breaks was observed in the DG after exposure to all concentrations of a single FQ, MARB, which showed a concentration-response trend. On the other hand, all FQs induced significant DNA strand breaks in the G (SSF<0), with the exception of the lowest SARA concentration (Fig. 3B).

### 3.6 Multivariate analysis

**Fig. 4:**
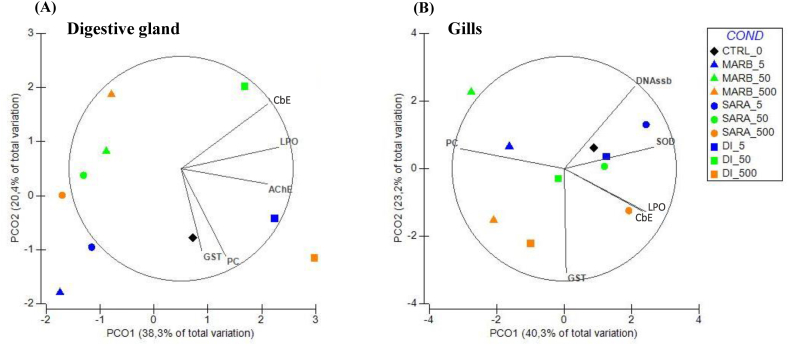
Centroid ordination diagrams (PCO) were generated for the S9 fractions of the digestive glands (panel A) and gills (panel B) in mussels. These diagrams depicted the ordination patterns for unexposed samples (0 ng/L) and those exposed to different concentrations (5, 50, and 500 ng/L) of marbofloxacin (MARB), sarafloxacin (SARA), and difloxacin (DI). Pearson correlation vectors (r > 0.75) are superimposed as supplementary variables, namely biochemical data: superoxide dismutase (SOD); glutathione peroxidase (GPx); glutathione-S-transferase (GST); carboxylesterase (CbE); lipid peroxidation (LPO); protein carbonylation (PC); acetylcholinesterase (AChE); DNA single-strand break (DNAssb).

Concerning the DG, The first principal component (PCO1), explaining 38.3 % of the total variance, clearly distinguished DI-exposed S9 fractions (positive side) from those exposed to MARB and SARA (negative side). Biomarkers such as CbE, LPO, AChE, GST, and PC significantly contribute to the variance in the positive direction of PCO1. Notably, CbE shows a strong correlation with 50 ng/L DI, and AChE with 5 ng/L DI. On the other hand, PCO2 accounts for 20.4 % of the total variance among conditions, differentiating the DG exposed to higher concentrations of all antibiotics (except DI_500) on the positive side from other concentrations and the control condition (CTRL_0) on the negative side (Fig. 4A).

In the context of gill analysis, PCO1, representing 40.3 % of the total variability, effectively discriminated S9 fractions exposed to all concentrations of SARA and 500 ng/L DI (on the positive side) from those exposed to MARB and other DI treatments. The key variables contributing to this distinction include SOD, CbE, LPO, and DNAssb. PCO2 (23.2 % of the total variability) distinctly separated the lowest and highest concentrations (5, 500 ng/L) of all tested FQs on the positive side from the lowest concentrations on the negative side. The variables primarily influencing the PCO2 axis were SOD, PC, and DNAssb (Fig. 4B).

## 4 Discussion

This *in vitro* study focused on examining the sub-cellular biochemical responses in the DG and G of the Mediterranean mussel *M. galloprovincialis* when exposed to ecologically relevant concentrations of three veterinary FQs, specifically MARB, SARA, and DI.

It is crucial to recognize that FQs have recently emerged as one of the primary residual antibiotics in aquatic environments and they may have broader and more complex environmental impacts beyond antibiotic resistance alone. Despite the frequent detection of FQs in aquatic environments, their specific effects on marine organisms have been relatively unexplored [36].

The significance and relevance of this study stem from its investigation into the toxicity of FQs at the cellular and subcellular levels in marine invertebrates, addressing the scarcity of information concerning their effects on non-target organisms, especially bivalve species. The concentrations selected for this study reflect those typically found in contaminated aquatic systems, ensuring that the range tested reflects realistic environmental exposures and, thus ecological relevance.

Utilizing the *in vitro* S9-based approach provides a reliable method for evaluating the toxicity of environmental pollutants, such as antibiotic pharmaceuticals, with the advantage of reducing time, costs, and animal usage. This approach serves as a valuable *screening* tool to identify potential interactions that may occur *in vivo*, identifying the most appropriate biomarkers to understand the cellular and molecular mechanisms underlying responses of important marine bivalve species, such as mussels, to these pollutants [37,38]. Specifically, we explored the effects of FQs on enzymatic antioxidant defenses, enzymatic detoxification mechanisms, and integrity of membrane lipids and proteins. Furthermore, we assessed their impact on neurotransmission and DNA integrity.

Enzymatic defenses against oxidative stress, such as superoxide dismutase (SOD) and glutathione peroxidase (GPx), are crucial for protecting cells against the overproduction of reactive oxygen species (ROS), potentially induced by environmental chemical stress. In this system, SOD plays a role in converting superoxide radicals (O_2_^−^) into hydrogen peroxide (H_2_O_2_). GPx is involved in converting H_2_O_2_ into water (H_2_O) and oxygen (O_2_). An excess release of reactive species may disrupt the balance of these antioxidants. FQs can stimulate the production of ROS, leading to an increase in singlet oxygen and superoxide anion quantities, potentially causing oxidative stress in the tissues [39,40].

In this study, both enzymes (SOD and GPx) exhibited a consistent pattern of overall decreasing activity in DG tissue when exposed to the tested FQs. As regards GPx activity reduction, it may be influenced by the activation of GST, discussed later in this study. GST uses GSH as a substrate, potentially depleting GSH levels and limiting its availability for GPx, which relies on GSH to neutralize peroxides [41]. This competition for GSH could explain the decreased GPx activity observed in this study.

A somewhat different trend emerged in G tissue, particularly in the case of SOD activity. When G fractions were exposed to MARB and DI, there was a general reduction in antioxidant activity. Conversely, exposure to SARA revealed an observable trend of increased SOD activity, implying a potential defensive response mechanism against this antibiotic.

Generally, a reduction of enzymatic antioxidant defense activity suggests potential alterations in the structure and functionality of the enzymatic protein. This observation was supported by Pan et al. [42], who presented evidence about the increased susceptibility to cellular oxidative stress induced by two FQs, such as ciprofloxacin and enrofloxacin. This susceptibility was closely linked to alterations in the conformation and functionality of the copper/zinc superoxide dismutase (Cu/Zn-SOD) molecule, which could be the consequence of the formation of a complex between FQs and the enzyme.

Alterations in the antioxidant system related to FQs antibiotics in aquatic organisms were supported by both *in vitro* and *in vivo* studies. For example, a similar previous *in vitro* study involving G and DG of *M. galloprovincialis*, exposed to ciprofloxacin (CIP), enrofloxacin (ENR), and danofloxacin (DAN), showed an overall reduction in SOD and GPx activity in both tissues [43]. As regards *in vivo* studies, the effects of FQs on the responses of the antioxidant system were reported on different tissues of the fish *Cirrhinus mrigala* [39]. The study revealed an elevated SOD activity and a reduction of CAT activity in G, liver, and kidney of fishes exposed to CIP. Conversely, Wang et al. [44] observed inhibition of SOD activity and activation of CAT in croaker *Pseudosciaena crocea* under norfloxacin (NOR) stress at environmentally relevant concentrations. Similarly, Jin et al. [45] showed a SOD activity decreases in the soil polychaete *Enchytraeus crypticus* exposed to NOR.

As regards biotransformation capacity, glutathione-S-transferase (GST) and carboxylesterase (CbE) are pivotal enzymes in the detoxification processes, belonging to phases II and I, respectively. GST catalyzes the conjugation of electrophilic substrates with glutathione (GSH), rendering xenobiotic compounds more water-soluble and facilitating their elimination. CbE catalyzes the hydrolysis of esters, contributing to the metabolism of various substances, including pharmaceutical products [46].

In this study, the G tissue showed GST activation along with a reduction in CbE activity. In contrast, no significant changes in the activities of either enzyme were observed in the DG. The elevated activity of GST in G of *M. galloprovincialis* aligns with the findings of Giannessi et al. [47], who observed increased levels following exposure to CIP and ENR, at concentrations of 50 and 500 ng/L. Furthermore, *in vivo* studies on fishes have indicated increased GST activity in species subjected to various pharmaceuticals, especially norfloxacin [48,49], and ciprofloxacin [50,51].

The increase in GST levels in organisms exposed to xenobiotics, including FQs, suggests a defensive response mechanism against these antibiotics and advancements in the detoxification process to counteract the generation of ROS and protect the cell. Furthermore, the increased GST activity may also reflect the role of this enzyme in the depuration metabolism. The elevation of GST levels indicates that cells are actively attempting to detoxify and eliminate FQs from the system, highlighting its function as a defensive mechanism [52].

About CbE, several *in vitro* studies assessing the inhibition of this enzyme family in mammals have been conducted. In mussels, they are recognized as valuable tools for examining drug interactions as CbE activity may be inhibited by certain types of therapeutic drugs [46].

In this study, inhibition of CbE activity was observed in the G of *M. galloprovincialis* exposed to MARB and DI. A decrease in CbE activity was observed in a large number of *in vitro* studies on aquatic species exposed to different pharmaceutical products [[53], [54], [55]]. The results of this study suggest that FQs might act as inhibitors of CbE in G of mussels. However, due to the scarcity of ecotoxicological studies addressing antibiotic metabolism in non-mammals, it was not feasible to make a meaningful comparison with existing literature data.

The specific responses observed in the tissues may be due not only to differences in bioaccumulation, concentration-dependent factors, and time-dependent patterns [56] but also to the existence of various isoenzymes of GST and CbE in those tissues. For instance, Fitzpatrick & Sheehan [57] demonstrated that GST isoenzymes display unique patterns specific to different tissues in mussels.

During a state of oxidative stress, the excessive ROS could react with macromolecules such as lipids and proteins due to their overproduction or to the inefficiency of antioxidant capacity, leading to alterations in cell functions [58]. The interaction between ROS and lipids, specifically the unsaturated fatty acids in cell membranes, can result in lipid peroxidation (LPO). Additionally, when interacting with proteins, they induce a mechanism of protein oxidation, namely protein carbonylation (PC). The accumulation of these oxidized protein aggregates, which cells find challenging to degrade, can result in impaired cellular function [59,60].

Despite the reduction in antioxidant enzyme activities noted in this study, no signs of cellular damage were observed. This finding suggests that a short exposure may not be enough to trigger membrane disruption, and it implies that mussels may have evolved alternative defense mechanisms to eliminate potential ROS and prevent their interaction with lipids.

The exception was made for PC levels in the G: in this tissue, exposure to MARB and DI led to an increase in PC levels in comparison to the unexposed tissue. The oxidative potential of FQs has been noted in various studies across different species. In a prior *in vitro* investigation [61], it was shown that FQs residues in bovine muscle led to substantial carbonylation in major muscular proteins, including sarcoplasmic and myofibrillary proteins. This observation extends to muscle proteins in adult earthworms (*Eisenia fetida*), as demonstrated in another study [62].

Authors [63,64] proposed a mechanism for protein carbonylation induced by fluoroquinolones (FQs). They indicate that this process is linked to the structural similarities between these compounds and quinones found in the mitochondrial electron transport chain (ETC). This hypothesis posits that FQs may function as inhibitors at the binding sites for transporting quinones within ETC complex II, a phenomenon also observed in plants. Disruption of the ETC can elevate oxidative stress by generating free radicals, which may subsequently lead to protein oxidation through direct interactions.

The acetylcholinesterase (AChE) enzyme plays a crucial role in the transmission of nerve impulses by catalyzing the hydrolysis of acetylcholine into choline and acetate. Inhibition in its activity results in the accumulation of acetylcholine, leading to a hyperpolarized state of the post-synaptic membrane and the disruption of nervous transmission. Consequently, neurotoxic compounds that inhibit AChE can induce significant dysfunction in aquatic organisms [65]. Furthermore, AChE is sensitive to various neurotoxic compounds, making it widely employed as an indicator of potential neurotoxicity [66].

In this study, AChE activity had a different response pattern between tissues: in DG have been observed a significant inhibition of its activity when exposed to all concentrations of MARB and SARA. Conversely, in G exposed fractions, no significant differences were observed compared to the control condition.

These results are consistent with an *in vitro* study indicating a decrease in AChE activity specifically in the DG of *M. galloprovicialis* following exposure to three distinct FQs, namely CIP, ENR, and DAN [43].

In silico docking studies and biochemical assessments using purified enzymes have demonstrated that certain derivatives of FQs exhibit potent inhibitory activity on AChE, indicating an affinity for the enzyme's active site. Additionally, synthesized compounds showed an increased trend in inhibitory activity, particularly those carrying electronegative functions at the *ortho* position of the phenyl group [67,68].

These findings suggest a potential impairment of neurotransmission due to FQs exposure in mussels’ DG, and imply the existence of isoforms in the G distinct from those found in the DG.

FQs antibiotics inhibit the activity of bacterial gyrase, a type II topoisomerase specific to prokaryotes. The eukaryotic counterpart, TOPO II, is a similar enzyme responsible for regulating DNA topology. When maintained at sufficient concentrations, there is a possibility that FQs could impede the function of isolated mammalian TOPO II enzymes *in vitro*, as indicated by Ref. [69]. This raises concerns regarding potential genotoxic effects in eukaryotes through DNA-intercalation, as highlighted in studies conducted *in vitro* and *in silico* [70]

In this study, almost all exposure treatments to FQs resulted in an increased frequency of strand breaks, thus causing a loss of DNA integrity in both G and DG.

Previous studies on various aquatic species have documented DNA damage following exposure to FQs. For instance, Jia et al. [71] observed DNA damage in zebrafish embryos exposed *in vivo* to CIP and ENR. Moreover, Liu et al. [72] reported significant DNA damage in goldfish (*Carassius auratus*) due to elevated levels of NOR, with the damage being concentration- and time-dependent. Additionally, Nunes et al. [73] found DNA damage in the crustacean *Daphnia magna* following exposure to CIP. All these findings suggest that if the mechanisms of DNA repair fail, exposure to FQs could lead to genotoxic damage in non-target species.

The PCO results demonstrated a clear separation between the responses in G and DG tissues, with G tissue showing a more pronounced biochemical response across multiple biomarkers, particularly in the antioxidant and detoxification systems. These data suggest that gills were generally more responsive and susceptible to the biochemical alterations induced by FQs compared to the digestive gland. This could depend on the fact that gills are in direct contact with the external environment and have a high surface area, which makes them more prone to immediate biochemical changes when exposed to contaminants. Moreover, G tissue contains a variety of receptors and ion channels involved in environmental sensing and regulation of physiological processes, which could make them more reactive to xenobiotics [74].

Furthermore, the PCO also highlighted distinct groupings of responses for each of the three FQs, suggesting individual toxicodynamic profiles. MARB and DI appeared to be more toxic than SARA, based on the overall pattern of enzymatic inhibition and cellular damage observed. Both MARB and DI led to significant reductions in antioxidant enzyme activities and increased protein carbonylation in G tissue, suggesting that they may be inducers of oxidative stress. On the other hand, SARA, while still affecting certain biomarkers such as SOD activity, induced fewer alterations in the detoxification and cellular damage pathways, indicating a comparatively lower toxicity.

## 5 Conclusions

Summarizing, the current investigation demonstrated that the Mediterranean mussel *M. galloprovincialis* exhibited tissue-specific subcellular responses when exposed to environmentally relevant concentrations of three veterinary fluoroquinolones MARB, SARA, and DI. Emerging contaminants like antibiotics particularly benefit from initial *in vitro* evaluations, as there is often limited experimental data on their effects. *In vitro* assessments, like those in the present study, enable a more targeted toxicological approach, allowing us to assess the sensitivity of different tissues, biomarkers, and signaling pathways.

This study revealed that each tested FQ induced specific biochemical responses, with MARB and DI showing more pronounced effects compared to SARA. The gills were particularly sensitive to oxidative stress and protein carbonylation, suggesting a heightened vulnerability to FQ-induced cellular damage. Conversely, the DG exhibited significant inhibition of AChE, suggesting an inhibitory activity driven by the parent compounds or biotransformation derivates. Furthermore, we observed DNA strand breaks in both tissues, highlighting the genotoxic potential of FQs and raising concerns about long-term genetic damage in marine organisms exposed to these pollutants.

The tissue-specific patterns identified in this study underscore the complexity of FQ toxicity and suggest that even at environmentally relevant concentrations, these antibiotics can compromise essential biochemical functions in marine bivalves. These findings are particularly relevant in light of the widespread use of FQs in veterinary medicine, emphasizing the need for further research on the chronic effects of low-level, long-term exposure in non-target species. Additionally, the study highlights the importance of including neurotoxicity and genotoxicity endpoints in future ecotoxicological assessments of antibiotics to better understand their full impact on marine ecosystems.

## CRediT authorship contribution statement

**J. Giannessi:** Writing – review & editing, Writing – original draft, Methodology, Investigation, Formal analysis, Data curation. **L. De Marchi:** Writing – review & editing, Investigation, Formal analysis, Data curation. **V. Meucci:** Writing – review & editing, Methodology, Investigation, Formal analysis. **L. Intorre:** Writing – review & editing, Supervision, Project administration, Funding acquisition, Conceptualization. **G. Monni:** Investigation, Formal analysis. **M. Baratti:** Writing – review & editing, Data curation. **C. Pretti:** Writing – review & editing, Validation, Supervision, Project administration, Funding acquisition, Conceptualization.

## Data and code availability

Data will be made available on request.

## Declaration of competing interest

The authors declare that they have no known competing financial interests or personal relationships that could have appeared to influence the work reported in this paper.

## References

[1] Du J., Liu Q., Pan Y., Xu S., Li H., Tang J. (2023). The research status, potential hazards and toxicological mechanisms of fluoroquinolone antibiotics in the environment. Antibiotics.

[2] Drlica K., Zhao X. (1997). DNA gyrase, topoisomerase IV, and the 4-quinolones. Microbiol. Mol. Biol. Rev..

[3] Pham T.D., Ziora Z.M., Blaskovich M.A. (2019). Quinolone antibiotics. Medchemcomm.

[4] Riviere J.E., Papich M.G. (2018). Veterinary Pharmacology and Therapeutics.

[5] Brar R.K., Jyoti U., Patil R.K., Patil H.C. (2020). Fluoroquinolone antibiotics: an overview. Adesh University Journal of Medical Sciences & Research.

[6] Robinson A.A., Belden J.B., Lydy M.J. (2005). Toxicity of fluoroquinolone antibiotics to aquatic organisms. Environ. Toxicol. Chem.: Int. J..

[7] Carvalho I.T., Santos L. (2016). Antibiotics in the aquatic environments: a review of the European scenario. Environ. Int..

[8] Janecko N., Pokludova L., Blahova J., Svobodova Z., Literak I. (2016). Implications of fluoroquinolone contamination for the aquatic environment—a review. Environ. Toxicol. Chem..

[9] de Ilurdoz M.S., Sadhwani J.J., Reboso J.V. (2022). Antibiotic removal processes from water & wastewater for the protection of the aquatic environment-a review. J. Water Proc. Eng..

[10] Felis E., Kalka J., Sochacki A., Kowalska K., Bajkacz S., Harnisz M., Korzeniewska E. (2020). Antimicrobial pharmaceuticals in the aquatic environment-occurrence and environmental implications. Eur. J. Pharmacol..

[11] Larsson D.G., Flach C.F. (2022). Antibiotic resistance in the environment. Nat. Rev. Microbiol..

[12] Bhatt S., Chatterjee S. (2022). Fluoroquinolone antibiotics: occurrence, mode of action, resistance, environmental detection, and remediation–A comprehensive review. Environ. Pollut..

[13] Baralla E., Demontis M.P., Dessì F., Varoni M.V. (2021). An overview of antibiotics as emerging contaminants: occurrence in bivalves as biomonitoring organisms. Animals.

[14] Nie X., Gu J., Lu J., Pan W., Yang Y. (2009). Effects of norfloxacin and butylated hydroxyanisole on the freshwater microalga Scenedesmus obliquus. Ecotoxicology.

[15] Carlsson G., Patring J., Kreuger J., Norrgren L., Oskarsson A. (2013). Toxicity of 15 veterinary pharmaceuticals in zebrafish (Danio rerio) embryos. Aquat. Toxicol..

[16] Bhatt S., Chatterjee S. (2022). Fluoroquinolone antibiotics: occurrence, mode of action, resistance, environmental detection, and remediation–A comprehensive review. Environ. Pollut..

[17] Schmerold I., van Geijlswijk I., Gehring R. (2023). European regulations on the use of antibiotics in veterinary medicine. Eur. J. Pharmaceut. Sci..

[18] Ding H.Z., Zeng Z.L., Fung K.F., Chen Z.L., Qiao G.L. (2001). Pharmacokinetics of sarafloxacin in pigs and broilers following intravenous, intramuscular, and oral single‐dose applications. J. Vet. Pharmacol. Therapeut..

[19] Ding H.Z., Yang G.X., Huang X.H., Chen Z.L., Zeng Z.L. (2008). Pharmacokinetics of difloxacin in pigs and broilers following intravenous, intramuscular, and oral single‐dose applications. J. Vet. Pharmacol. Therapeut..

[20] Goudah A., Hasabelnaby S. (2011). The disposition of marbofloxacin after single dose intravenous, intramuscular and oral administration to Muscovy ducks. J. Vet. Pharmacol. Therapeut..

[21] Du J., Liu Q., Pan Y., Xu S., Li H., Tang J. (2023). The research status, potential hazards and toxicological mechanisms of fluoroquinolone antibiotics in the environment. Antibiotics.

[22] Maghsodian Z., Sanati A.M., Mashifana T., Sillanpää M., Feng S., Nhat T., Ramavandi B. (2022). Occurrence and distribution of antibiotics in the water, sediment, and biota of freshwater and marine environments: a review. Antibiotics.

[23] Van Doorslaer X., Dewulf J., Van Langenhove H., Demeestere K. (2014). Fluoroquinolone antibiotics: an emerging class of environmental micropollutants. Sci. Total Environ..

[24] Hubbard S.A., Brooks T.M., Gonzalez L.P., Bridges J.W. (1985). Comparative Genetic Toxicology: the Second UKEMS Collaborative Study.

[25] Lowry O.H., Rosebrough N.J., Farr A.L., Randall R.J. (1951). Protein measurement with the Folin phenol reagent. J. Biol. Chem..

[26] Pretti C., Aretini P., Lessi F., Freitas R., Barata C., De Marchi L., Baratti M. (2023). Gene expression and biochemical patterns in the digestive gland of the mussel Mytilus galloprovincialis (Lamarck, 1819) exposed to 17α-ethinylestradiol. Aquat. Toxicol..

[27] Badary O.A., Abdel-Maksoud S., Ahmed W.A., Owieda G.H. (2005). Naringenin attenuates cisplatin nephrotoxicity in rats. Life Sci..

[28] Magnani L., Gaydou E.M., Hubaud J.C. (2000). Spectrophotometric measurement of antioxidant properties of flavones and flavonols against superoxide anion. Anal. Chim. Acta.

[29] Habig W.H., Pabst M.J., Jakoby W.B. (1976). Glutathione S-transferase AA from rat liver. Arch. Biochem. Biophys..

[30] Hosokawa M., Satoh T. (2001). Measurement of carboxylesterase (CES) activities. Current protocols in toxicology.

[31] Ohkawa H., Ohishi N., Yagi K. (1979). Assay for lipid peroxides in animal tissues by thiobarbituric acid reaction. Anal. Biochem..

[32] Mesquita C.S., Oliveira R., Bento F., Geraldo D., Rodrigues J.V., Marcos J.C. (2014). Simplified 2, 4-dinitrophenylhydrazine spectrophotometric assay for quantification of carbonyls in oxidized proteins. Anal. Biochem..

[33] Ellman G.L., Courtney K.D., Andres Jr V., Featherstone R.M. (1961). A new and rapid colorimetric determination of acetylcholinesterase activity. Biochem. Pharmacol..

[34] Schröder H.C., Batel R., Schwertner H., Boreiko O., Müller W.E. (2006). Fast micromethod DNA single-strand-break assay. DNA Repair Protocols: Mammalian Systems.

[35] Clarke K.R., Somerfield P.J., Gorley R.N. (2008). Testing of null hypotheses in exploratory community analyses: similarity profiles and biota-environment linkage. J. Exp. Mar. Biol. Ecol..

[36] Shen M., Hu Y., Zhao K., Li C., Liu B., Li M., Zhong S. (2023). Occurrence, bioaccumulation, metabolism and ecotoxicity of fluoroquinolones in the aquatic environment: a review. Toxics.

[37] Khan B., Burgess R.M., Fogg S.A., Cantwell M.G., Katz D.R., Ho K.T. (2018). Cellular responses to in vitro exposures to β-blocking pharmaceuticals in hard clams and Eastern oysters. Chemosphere.

[38] Parolini M., Binelli A., Cogni D., Riva C., Provini A. (2009). An in vitro biomarker approach for the evaluation of the ecotoxicity of non-steroidal anti-inflammatory drugs (NSAIDs). Toxicol. Vitro.

[39] Ramesh M., Sujitha M., Anila P.A., Ren Z., Poopal R.K. (2021). Responses of Cirrhinus mrigala to second‐generation fluoroquinolone (ciprofloxacin) toxicity: assessment of antioxidants, tissue morphology, and inorganic ions. Environ. Toxicol..

[40] Qin P., Liu R. (2013). Oxidative stress response of two fluoroquinolones with catalase and erythrocytes: a combined molecular and cellular study. J. Hazard Mater..

[41] Pei J., Pan X., Wei G., Hua Y. (2023). Research progress of glutathione peroxidase family (GPX) in redoxidation. Front. Pharmacol..

[42] Pan X., Qin P., Liu R., Li J., Zhang F. (2016). Molecular mechanism on two fluoroquinolones inducing oxidative stress: evidence from copper/zinc superoxide dismutase. RSC Adv..

[43] Giannessi J., De Marchi L., Meucci V., Intorre L., Monni G., Baratti M., Pretti C. (2023). Subcellular tissue-specific responses of Mytilus galloprovincialis to fluoroquinolone antibiotics. Environ. Toxicol. Pharmacol..

[44] Wang X., Hu M., Gu H., Zhang L., Shang Y., Wang T., Wang Y. (2020). Short-term exposure to norfloxacin induces oxidative stress, neurotoxicity and microbiota alteration in juvenile large yellow croaker Pseudosciaena crocea. Environ. Pollut..

[45] Jin M.K., Zhang Q., Zhao W.L., Li Z.H., Qian H.F., Yang X.R., Liu H.J. (2022). Fluoroquinolone antibiotics disturb the defense system, gut microbiome, and antibiotic resistance genes of Enchytraeus crypticus. J. Hazard Mater..

[46] Solé M., Sanchez-Hernandez J.C. (2018). Elucidating the importance of mussel carboxylesterase activity as exposure biomarker of environmental contaminants of current concern: an in vitro study. Ecol. Indicat..

[47] Giannessi J., De Marchi L., Meucci V., Intorre L., Monni G., Baratti M., Pretti C. (2023). Subcellular tissue-specific responses of Mytilus galloprovincialis to fluoroquinolone antibiotics. Environ. Toxicol. Pharmacol..

[48] Andrieu M., Rico A., Phu T.M., Phuong N.T., Van den Brink P.J. (2015). Ecological risk assessment of the antibiotic enrofloxacin applied to Pangasius catfish farms in the Mekong Delta, Vietnam. Chemosphere.

[49] Bartoskova M., Dobsikova R., Stancova V., Pana O., Zivna D., Plhalova L., Marsalek P. (2014). Norfloxacin—toxicity for zebrafish (Danio rerio) focused on oxidative stress parameters. BioMed Res. Int..

[50] Plhalova L., Zivna D., Bartoskova M., Blahova J., Sevcikova M., Skoric M., Svobodova Z. (2014). The effects of subchronic exposure to ciprofloxacin on zebrafish (Danio rerio). Neuroendocrinol. Lett..

[51] Ramesh M., Sujitha M., Anila P.A., Ren Z., Poopal R.K. (2021). Responses of Cirrhinus mrigala to second‐generation fluoroquinolone (ciprofloxacin) toxicity: assessment of antioxidants, tissue morphology, and inorganic ions. Environ. Toxicol..

[52] Strange R.C., Jones P.W., Fryer A.A. (2000). Glutathione S-transferase: genetics and role in toxicology. Toxicol. Lett..

[53] Nos D., Navarro J., Saiz E., Sanchez-Hernandez J.C., Solé M. (2020). Tetrabromobisphenol A inhibits carboxylesterase activity of marine organisms from different trophic levels. Chemosphere.

[54] Solé M., Sanchez-Hernandez J.C. (2018). Elucidating the importance of mussel carboxylesterase activity as exposure biomarker of environmental contaminants of current concern: an in vitro study. Ecol. Indicat..

[55] Solé M., Freitas R., Rivera-Ingraham G. (2021). The use of an in vitro approach to assess marine invertebrate carboxylesterase responses to chemicals of environmental concern. Environ. Toxicol. Pharmacol..

[56] Benito D., Niederwanger M., Izagirre U., Dallinger R., Soto M. (2017). Successive onset of molecular, cellular and tissue-specific responses in midgut gland of Littorina littorea exposed to sub-lethal cadmium concentrations. Int. J. Mol. Sci..

[57] Fitzpatrick P.J., Sheehan D. (1993). Separation of multiple forms of glutathione S-transferase from the blue mussel, Mytilus edulis. Xenobiotica.

[58] Regoli F., Giuliani M.E. (2014). Oxidative pathways of chemical toxicity and oxidative stress biomarkers in marine organisms. Mar. Environ. Res..

[59] Cunha M., Silva M.G., De Marchi L., Morgado R.G., Esteves V.I., Meucci V., Freitas R. (2023). Toxic effects of a mixture of pharmaceuticals in Mytilus galloprovincialis: the case of 17α-ethinylestradiol and salicylic acid. Environ. Pollut..

[60] Suzuki Y.J., Carini M., Butterfield D.A. (2010). Protein carbonylation. Antioxidants Redox Signal..

[61] Márquez-Lázaro J., Méndez-Cuadro D., Rodríguez-Cavallo E. (2020). Residues of fluoroquinolone antibiotics induce carbonylation and reduce in vitro digestion of sarcoplasmic and myofibrillar beef proteins. Foods.

[62] Márquez-Lázaro J., Díaz-Pineda K., Méndez-Cuadro D., Rodríguez-Cavallo E. (2021). Fluoroquinolone antibiotics and organophosphate pesticides induce carbonylation on Eisenia fetida muscle proteins. Sci. Total Environ..

[63] Gomes M.P., Gonçalves C.A., de Brito J.C.M., Souza A.M., da Silva Cruz F.V., Bicalho E.M., Garcia Q.S. (2017). Ciprofloxacin induces oxidative stress in duckweed (Lemna minor L.): implications for energy metabolism and antibiotic-uptake ability. J. Hazard Mater..

[64] Nunes B., Veiga V., Frankenbach S., Serôdio J., Pinto G. (2019). Evaluation of physiological changes induced by the fluoroquinolone antibiotic ciprofloxacin in the freshwater macrophyte species Lemna minor and Lemna gibba. Environ. Toxicol. Pharmacol..

[65] Fulton M.H., Key P.B. (2001). Acetylcholinesterase inhibition in estuarine fish and invertebrates as an indicator of organophosphorus insecticide exposure and effects. Environ. Toxicol. Chem.: Int. J..

[66] Matozzo V., Tomei A., Marin M.G. (2005). Acetylcholinesterase as a biomarker of exposure to neurotoxic compounds in the clam Tapes philippinarum from the Lagoon of Venice. Mar. Pollut. Bull..

[67] Mansha M., Taha M., Ullah N. (2021). The design of fluoroquinolone-based cholinesterase inhibitors: synthesis, biological evaluation and in silico docking studies. Arab. J. Chem..

[68] Bautista-Aguilera Ó.M., Ismaili L., Chioua M., Andrys R., Schmidt M., Bzonek P., Marco-Contelles J. (2020). Acetylcholinesterase inhibition of diversely functionalized quinolinones for Alzheimer's disease therapy. Int. J. Mol. Sci..

[69] Williams G.M., Brunnemann K.D., Smart D.J., Molina D., Jeffrey A.M., Duan J.D., Schmuck G. (2013). Relationship of cellular topoisomerase IIα inhibition to cytotoxicity and published genotoxicity of fluoroquinolone antibiotics in V79 cells. Chem. Biol. Interact..

[70] Bhattacharya P., Mukherjee S., Mandal S.M. (2020). Fluoroquinolone antibiotics show genotoxic effect through DNA-binding and oxidative damage. Spectrochim. Acta Mol. Biomol. Spectrosc..

[71] Jia D., You X., Tang M., Lyu Y., Hu J., Sun W. (2023). Single and combined genotoxicity of metals and fluoroquinolones to zebrafish embryos at environmentally relevant concentrations. Aquat. Toxicol..

[72] Liu J., Lu G., Wu D., Yan Z. (2014). A multi-biomarker assessment of single and combined effects of norfloxacin and sulfamethoxazole on male goldfish (Carassius auratus). Ecotoxicol. Environ. Saf..

[73] Nunes B., Leal C., Rodrigues S., Antunes S.C. (2018). Assessment of ecotoxicological effects of ciprofloxacin in Daphnia magna: life-history traits, biochemical and genotoxic effects. Water Sci. Technol..

[74] Liao X., Liu Y., Han T., Yang M., Liu W., Wang Y., Lu Z. (2022). Full-length transcriptome sequencing reveals tissue-specific gene expression profile of mangrove clam geloina erosa. Front. Physiol..

