# Supplementary material for: Veterinary fluoroquinolones as emerging contaminants in marine environments: In vitro study of biochemical responses in subcellular fractions of the Mediterranean mussel (Mytilus galloprovincialis)
Source: Heliyon. 2024 Nov 17;10(22):e40467. doi: 10.1016/j.heliyon.2024.e40467 (PMC11617211; doi:10.1016/j.heliyon.2024.e40467)
Supplement: Multimedia component 1 [file mmc1.docx]

**Captions**

**Figure S1**: biochemical parameters evaluated on unexposed S9-fractions, specifically SOD, GPx, GST, CbE, LPO, PC, and AChE. Each parameter was assessed at 30 and 60 minutes with 0.5 or 1 mg of total protein. The results obtained were compared to identify the maximum values achieved for each assay, and these values were then compared against all other outcomes. A one-way ANOVA, followed by a post-test, was performed to statistically analyze the data. Statistically significant differences between the highest value result and all the other tested conditions were denoted with asterisks: *p < 0.05; **p < 0.01; *** p < 0.001; ****p<0.0001.
