# Supplementary figures and images for: Veterinary fluoroquinolones as emerging contaminants in marine environments: In vitro study of biochemical responses in subcellular fractions of the Mediterranean mussel (Mytilus galloprovincialis)
Source: Heliyon. 2024 Nov 17;10(22):e40467. doi: 10.1016/j.heliyon.2024.e40467 (PMC11617211; doi:10.1016/j.heliyon.2024.e40467)

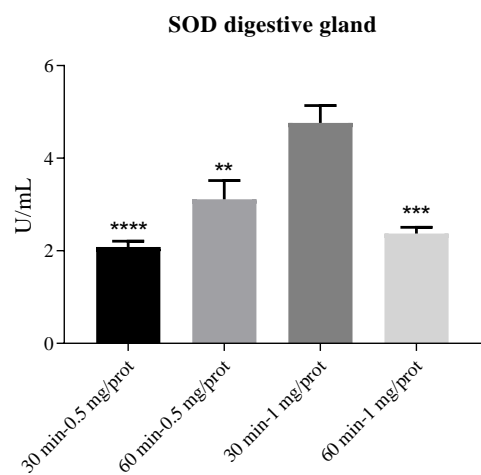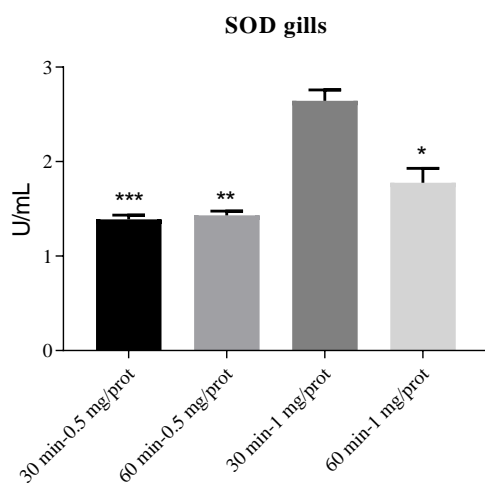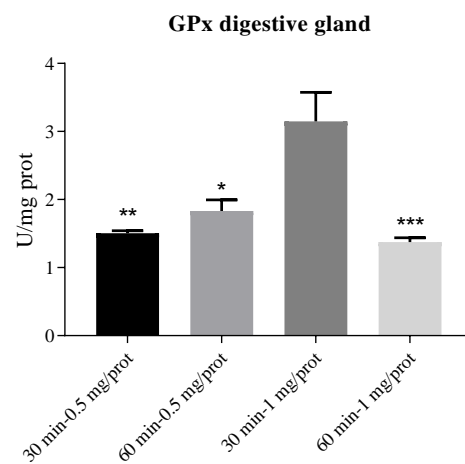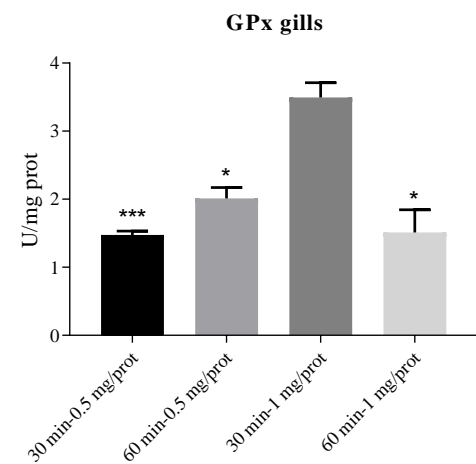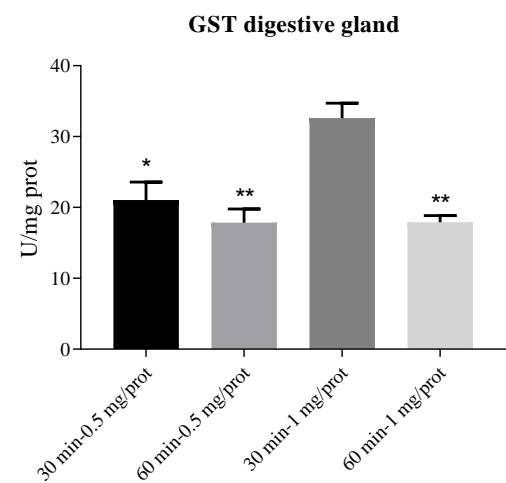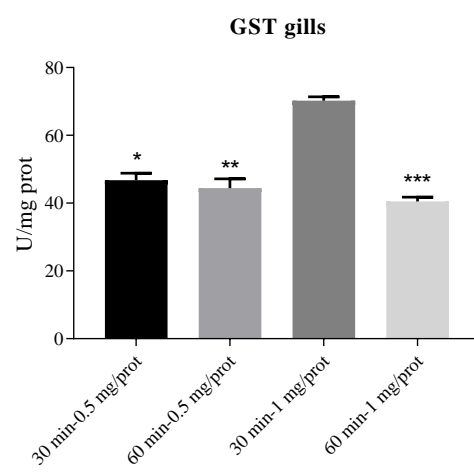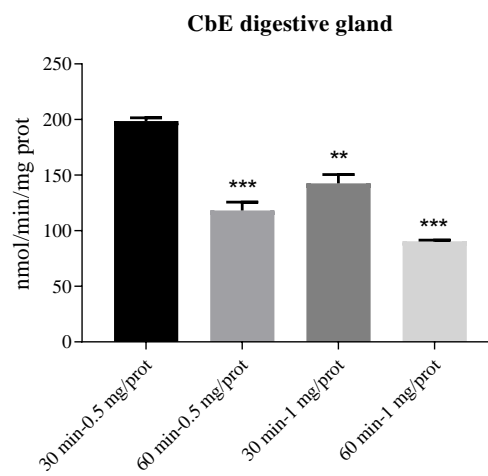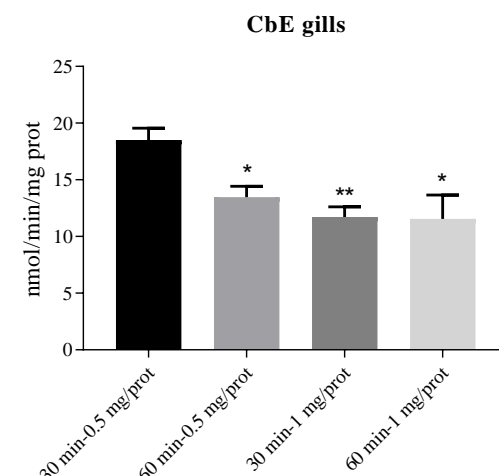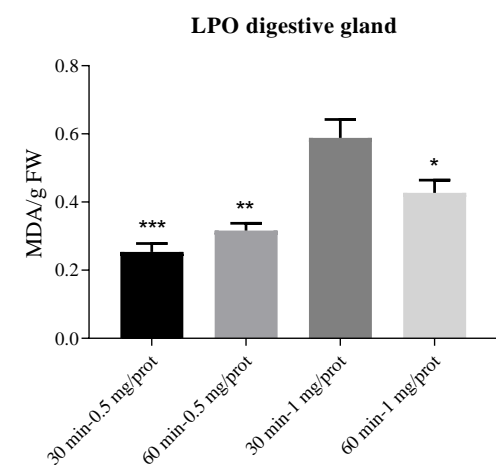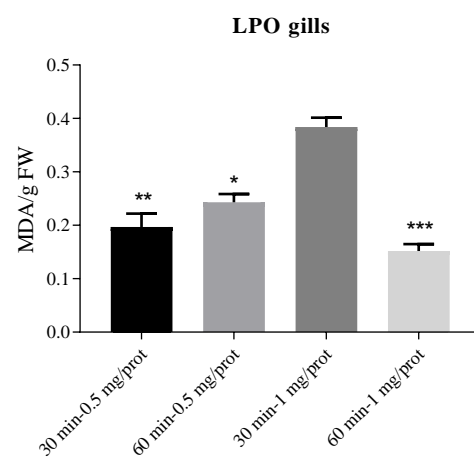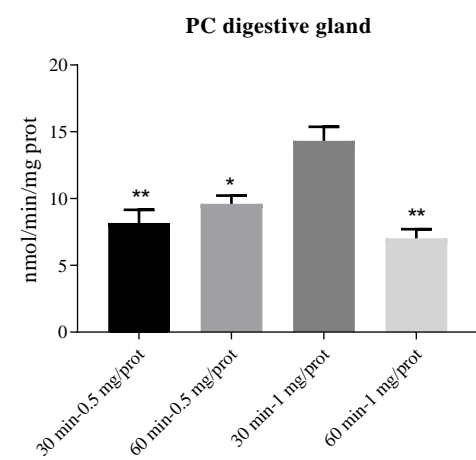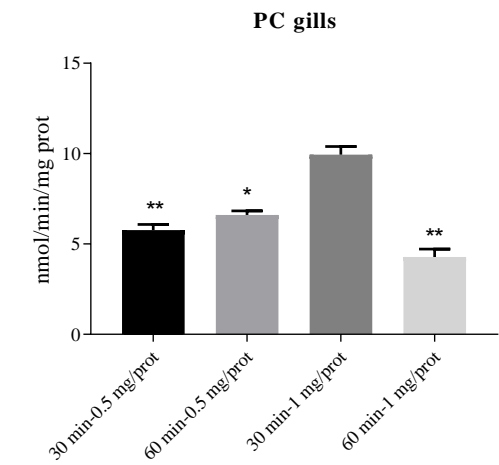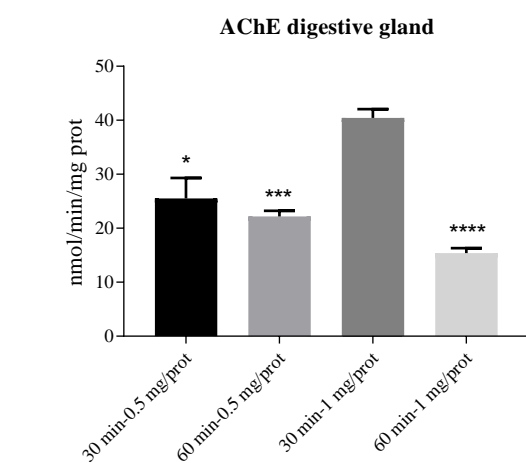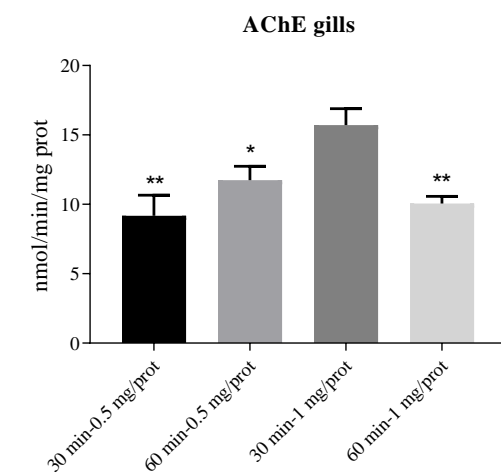

Supplement: Multimedia component 2 [file mmc2.pdf]
